# The Level of D-Dimer Is Positively Correlated With the Severity of *Mycoplasma pneumoniae* Pneumonia in Children

**DOI:** 10.3389/fcimb.2021.687391

**Published:** 2021-07-15

**Authors:** Yan Zheng, Lingling Hua, Qiannan Zhao, Mengyao Li, Meixia Huang, Yunlian Zhou, Yingshuo Wang, Zhimin Chen, Yuanyuan Zhang

**Affiliations:** ^1^ Department of Pulmonology, Children’s Hospital Zhejiang University School of Medicine, National Clinical Research Center for Child Health, Hangzhou, China; ^2^ Department of Pediatrics, The Quzhou Affiliated Hospital of Wenzhou Medical University, Quzhou People’s Hospital, Quzhou, China; ^3^ Department of Pediatrics, Ningbo Women and Children’s Hospital, Ningbo, China

**Keywords:** *Mycoplasma pneumoniae*, pneumonia, D-dimer, children, severity

## Abstract

**Objective:**

*Mycoplasma pneumoniae* pneumonia (MPP) is an important disease in children. Studies have demonstrated that the levels of D-dimer are elevated in some children with MPP, especially those with thrombotic complications. However, the potential association between MPP and D-dimer remains unclear. In our study, we sought to explore the relationship between the levels of plasma D-dimer and clinical characteristics of MPP patients.

**Methods:**

Retrospective analysis was conducted on 356 patients who were hospitalized in our hospital for MPP between January 1, 2017, and December 31, 2019. According to the peak value of D-dimer, patients were divided into three groups: the normal group (D-dimer<0.55 mg/L), the mild-moderately elevated group (D-dimer 0.55–5.5 mg/L) and the severely elevated group (D-dimer >5.5 mg/L). The demographic and clinical information, radiological findings, laboratory data, and treatments of patients were compared among different groups.

**Results:**

106 patients were in the normal group, 204 patients were in the mild-moderately elevated group, and 46 patients were in the severely elevated group. More severe clinical and radiographic manifestations, longer length of fever, hospital stay and antibiotic therapy duration, higher incidences of extra-pulmonary complications, refractory MPP (RMPP), severe MPP (SMPP) were found in the elevated group, when compared with the normal group (*P*<0.01). Meanwhile, we found that the percentage of neutrophil (N%) and CD_8_^+^ lymphocyte (CD_8_^+^%), C-reactive protein (CRP), lactate dehydrogenase (LDH), interleukin (IL)-6, IL-10, and interferon-gamma (IFN-γ) trended higher with increasing D-dimer, whereas the percentage of lymphocyte (L%) and prealbumin (PAB) trended lower (*P*<0.01). In addition, the proportions of patients requiring oxygen therapy, glucocorticoid, bronchoscopy, immunoglobulin use, thoracentesis, or ICU admission were significantly higher in the severely elevated group than those in the other two groups (*P*<0.01). Correlation analysis showed that N%, L%, CRP, LDH, IL-10, length of fever, length of stay, and length of antibiotic therapy had strong correlations with the level of D-dimer.

**Conclusions:**

MPP patients with higher levels of D-dimer had more severe clinical manifestations and needed longer duration of treatment, which might be closely related to the severity of lung inflammation after MP infection.

## Introduction

*Mycoplasma pneumoniae* (MP) is one of the most important microorganisms that caused community-acquired pneumonia (CAP) in children (Jain et al., 2015; Meyer Sauteur et al., 2020). It may be responsible for about 4% to 8% of CAP during periods of endemicity, whereas it could cause up to 20% to 40% of CAP in the general population during epidemics (Waites et al., 2017). In general, *Mycoplasma pneumoniae* pneumonia (MPP) is recognized as a self-limited disease, but recently, some researchers reported that it could cause pulmonary and extra-pulmonary complications, or even life-threatening situations, such as necrotizing pneumonitis, myocarditis, hemolytic anemia, acute pancreatitis, and so on (Poddighe, 2018). Furthermore, several recent studies show that MPP patients could have concurrent pulmonary embolism, cerebral infarction, spleen infarction, and other systemic thrombotic diseases (Park et al., 2012; Kang et al., 2016; Mélé and Turc, 2018; Chen et al., 2020). The pathogenic mechanisms of MP infection may be related to the cytoadherence, intracellular localization, cytotoxicity, inflammation, and so on, which is still elusive (Waites and Talkington, 2004).

D-dimer is a soluble fibrin degradation product that results from the ordered breakdown of thrombi by the fibrinolytic system (Weitz et al., 2017). Numerous studies have reported that elevated D-dimer is associated with thrombotic diseases (Goldenberg et al., 2004; Nowak-Göttl et al., 2004). Consequently, D-dimer has been extensively used for the diagnosis of venous thromboembolism (VTE) (Adam et al., 2009). Recently, it has also been reported that the levels of D-dimer are related to the severity of coronavirus disease 2019 (COVID-19) (Sakka et al., 2020). However, few people had concentrated on the relationship between clinical manifestations of MPP and D-dimer.

In this study, we retrospectively analyzed the clinical characteristics of MPP patients with different degrees of D-dimer who were hospitalized in our hospital between January 1, 2017, and December 31, 2019, and explore the relationship between the levels of D-dimer and clinical characteristics of MPP patients.

## Methods

### Study Population

In this study, we retrospectively collected the clinical data of patients with MMP who were admitted to Children’s hospital, Zhejiang University School of Medicine between January 1, 2017, and December 31, 2019. The criteria for enrolling patients were as follows: (1) signs and symptoms indicative of CAP, including fever, cough, abnormal lung auscultation and new infiltrate(s) on chest radiograph; (2) had both positive results for MP RNA polymerase chain reaction (PCR) tests and positive results for MP-IgM (Witmer et al., 2007; Waites et al., 2017). Exclusion criteria were the follows (Zhang et al., 2014; Jin et al., 2018): (1) had positive results for other pathogens; (2) had received corticosteroids, intravenous immunoglobulin (IVIG), and anticoagulation therapy before admission; (3) with underlying diseases, such as chronic cardiac and pulmonary disease, rheumatic diseases, and immunodeficiency; (4) had incomplete medical records.

### Data Collection

Demographic and clinical information, radiological findings, laboratory data, such as blood routine test, C-reactive protein (CRP), lactate dehydrogenase (LDH), D-dimer, prealbumin (PAB), subpopulations of T lymphocytes, immunoglobulins, cytokines were retrospectively collected from all patients by reviewing their electronic medical records. During the hospitalization, clinical signs and symptoms of patients were obtained, including body temperature, respiratory rates, extra-pulmonary complications (Poddighe, 2018,) (Witmer et al., 2007; Zhou et al., 2014), and so on. All patients underwent chest radiography during the illness, confirming unequivocal focal or segmental infiltration with or without pleural effusion. Although patients had progressive symptoms, suspected complications, clinical deterioration, or persistent fever after appropriate antibiotic therapy, chest CT scans were performed. The large lesion was defined as the extent of infiltration on chest imaging more than one third of the lung (Uehara et al., 2011).

Severe MPP (SMPP) was defined as MPP with any one of the follows: (1) a poor general condition; (2) fastidium or dehydration; (3) disturbance of consciousness; (4) an increased respiratory rate (infants > 70 breaths/min and older children > 50 breaths/min); (5) dyspnea; (6) cyanosis; (7) extent of infiltration on chest X-ray ≥2/3 of one lung or multilobe involvement; (8) extra-pulmonary complications; (9) pleural effusion; (10) oxygen saturation in room air ≤92% (Subspecialty Group of Respiratory Diseases, 2013). Refractory MPP (RMPP) was diagnosed based on the presence of persistent fever and clinical, as well as radiological deterioration after azithromycin treatment for 7 days or longer (Tamura et al., 2008; Subspecialty Group of Respiratory Diseases, 2013). The indications for oxygen therapy, bronchoscopy, glucocorticoid, and mechanical ventilation were evaluated according to the guidelines for management of community-acquired pneumonia in children in China (Subspecialty Group of Respiratory Diseases, 2013).

### Sample Detection

Nasopharyngeal aspirate/swab specimens were routinely tested within 24 hours after admission. Peripheral blood samples were obtained on admission for detecting the laboratory data, and the abnormal values of D-dimer and inflammatory indicators, such as WBC, CRP, cytokines, and so on, were detected every 3 to 5 days thereafter.

Serum cytokines were routinely measured because this would be, to some extent, helpful for therapy decisions, and the costs were not expensive. We detected the concentrations of interleukin (IL)-2, IL-4, IL-6, IL-10, tumor necrosis factor-alpha (TNF-α), and interferon-gamma (IFN-γ) in serum by a CBA HumanTh1/Th2 Cytokine Kit II (BD Biosciences, San Diego, CA, USA). The value of D-dimer was determined *via* INNOVANCE D-dimer (SIEMENS, Marburg, Germany), the normal value was less than 0.55 mg/L. The MP IgM was determined *via* a specific Anti-MP ELISA assay (EUROIMMUN, Luebeck, Germany), the absorbance above 1.1 was determined positive. MP RNA detection was performed on an ABI 7500 detection system *via* SAT-MP Assay Kit (Rendu Biotechnology Co., Ltd, Shanghai, China). All steps were performed according to the manufacturer’s instructions and previous studies (Zhang et al., 2016; Li et al., 2017).

### Ethics

The study was approved by the ethics committee of the Children’s Hospital, Zhejiang University School of Medicine (2019-RIB-058). And the data from patients were collected anonymously.

### Statistical Analysis

SPSS 20.0 (IBM Corp., Armonk, NY, USA) was used for statistical analysis. Continuous data are shown as median (25th to 75th percentile). Categorical data were shown as number (%). The Kruskal-Wallis-H(K-W-H) method was used to compare differences in continuous variables among multiple groups. The Mann-Whitney U-test was used to compare differences in continuous variables between two groups. Pearson’s chi-square test was used to analyze differences between categorical variables. Spearman rank-correlation coefficients were used to describe the association between different variables and D-dimer. Statistical significance was defined as P<0.05.

## Results

### General Information of Patients

From January 1, 2017, to December 31, 2019, a total of 356 patients admitted to our hospital for MPP were enrolled in the study. All patients had positive MP PCR tests and serological detection. According to the peak value of D-dimer (Huang et al., 2021), they were divided into three groups, the normal group (D-dimer <0.55 mg/L, n=106), the mild-moderately elevated group (D-dimer 0.55–5.5 mg/L, n=204) and the severely elevated group (more than 10 times higher than the normal range, D-dimer >5.5 mg/L, n=46). As shown in **Table 1**, the median age in the normal group was 4.1 years (range, 2.1–6.1), younger than that in the mild-moderately elevated group 5.8 years (range, 4.3–7.0) and the severely elevated group 6.1 years (range, 4.6–8.0) (*P*<0.01), but no difference was found in gender distribution (*P*>0.05).

**Table 1:** Demographic and clinical characteristics of patients with MPP.

Clinical information	Normal group (n=106)	Mild-moderately elevated group (n=204)	Severely elevated group (n=46)	*P*-value
Sex (male/female)	56/50	91/113	21/25	0.397
Age, years	4.1 (2.1~6.1)	5.8 (4.3~7.0) ^a#^	6.1 (4.6~8.0) ^b#^	0.000
Clinical presentation, n (%)				
Fever	100 (94.3%)	203 (99.5%) ^a#^	46 (100.0%) ^b#^	0.010
Cough	106 (100.0%)	204 (100.0%)	46(100.0%)	1.000
Wheezing	34 (32.1%)	25 (12.3%) ^a#^	3 (6.5%) ^b#^	0.000
Chest pain	2 (1.9%)	3 (1.5%)	1 (2.2%)	1.000
Extra-pulmonary complications, n (%)	14 (13.2%)	72 (35.3%) ^a#^	33 (71.7%) ^b#,c#^	0.000
Digestive system	7 (6.6%)	45 (22.1%)	12 (26.1%)	
Cardiovascular system	2 (1.9%)	9 (4.4%)	5 (10.9%)	
Neurologic system	2 (1.9%)	4 (2.0%)	2 (4.3%)	
Hematologic system	1 (0.9%)	2 (1.0%)	3 (6.5%)	
Skin and Mucosae	1 (0.9%)	3 (1.5%)	0 (0.0%)	
Multi-systems	1 (0.9%)	9 (4.4%)	11 (23.9%)	
Length of fever, days	7.3±3.9	10.2±3.1 ^a#^	14.5±4.8 ^b#,c#^	0.000
Length of stay, days	6.0 (4.0~7.0)	7.0 (5.0~10.0) ^a#^	13.0 (10.0~18.0) ^b#,c#^	0.000
Length of antibiotic therapy days	10.5 (8.0~13.3)	12.0 (10.0~16.0) ^a#^	19.0 (15.0~25.0) ^b#,c#^	0.000
RMPP, n (%)	7 (6.6%)	85 (41.7%) ^a#^	40 (87.0%) ^b#,c#^	0.000
SMPP, n (%)	68 (64.2%)	176 (86.3%) ^a#^	46 (100.0%) ^b#,c#^	0.000

Data are presented as median (25^th^~75^th^ percentile), or number (percentage). ^#^P < 0.01; ^a^compared between normal group and mild-moderately elevated group; ^b^compared between normal group and severely elevated group; ^c^compared between mild-moderately elevated group and severely elevated group.

Digestive system complications: hepatic impairment, hepatomegaly.

Cardiovascular system complications: myocardial damage, pericardial effusion, Kawasaki disease.

Neurologic system complications: encephalitis, Guillain–Barre’s syndrome.

Hematologic system complications: anemia, cytopenia.

Skin and Mucosae complications: unspecific rashes, Stevens-Johnson syndrome.

RMPP, Refractory mycoplasma pneumonia pneumonia; SMPP, Severe mycoplasma pneumonia pneumonia.

### Clinical Characteristics of Patients

The most common symptoms of MPP were cough (100.0%) and fever (98.0%). Chest pain (1.7%) was rare, and 62 patients (17.4%) presented with wheezing. As shown in **Table 1**, the higher incidence of fever and the lower incidence of wheezing were found in the mild-moderately or severely elevated group than those in the normal group (*P*<0.01). We also found that the total length of fever, the total length of antibiotic therapy, the length of stay, and the incidences of RMPP and SMPP were significantly different among the three groups (*P*<0.01). Interestingly, there were 14 patients (13.2%) with extra-pulmonary complications in the normal group, 72 patients (35.3%) in the mild-moderately elevated group, and 33 patients (71.7%) in the severely elevated group, which showed significant differences among these three groups (*P*<0.01). Moreover, the total length of fever, the total length of antibiotic therapy, the length of stay, and the incidence of extra-pulmonary complications, RMPP, SMPP were increased with the level of D-dimer (*P*<0.01).

### Laboratory Findings of Patients

Laboratory findings of patients on admission were shown in **Table 2**. Besides D-dimer, the WBC, percentage of peripheral neutrophils (N%) and lymphocytes (L%), platelet (PLT), CRP, LDH, PAB, percentage of CD_4_^+^ and CD_8_^+^ T lymphocytes (CD_4_^+^%, CD_8_^+^%), IgA, IL-6, IL-10, TNF-α, IFN-γ also differed significantly among the three groups (*P*<0.01), but there were no statistically significant differences in hemoglobin, percentage of CD_3_^+^ T lymphocytes (CD_3_^+^%), IL-2, IL-4, IgG, IgM, and IgE (*P*>0.05). Furthermore, we found that N%, CRP, LDH, CD_8_^+^%, IL-6, IL-10, and IFN-γ were significantly increased in the elevated group, especially in the severely elevated group than those in the normal group, which were in line with the levels of D-dimer; whereas the L% and PAB decreased with the levels of D-dimer.

**Table 2:** Laboratory findings of patients with MPP on admission.

Laboratory information	Normal group (n=106)	Mild-moderately elevated group (n=204)	Severely elevated group (n=46)	*P*-value
Routine blood test				
White blood cell (×10^9^/L)	7.72 (6.28~9.79)	7.33 (5.79~9.36)	8.22 (6.72~10.65) ^c*^	0.048
Neutrophil, %	57.7 (43.5~65.2)	65.4 (55.9~71.8) ^a#^	75.9 (66.8~81.2) ^b#,c#^	0.000
Lymphocyte, %	31.2 (25.9~44.8)	24.7 (18.9~32.5) ^a#^	17.2 (12.6~25.7) ^b#,c#^	0.000
Hemoglobin, g/L	122 (113~128)	122 (116~129)	119 (114~129)	0.197
Platelet (×10^9^/L)	336 (267~405)	288 (222~361) ^a#^	262 (197~336) ^b#^	0.000
CRP, mg/L	3.4 (0.5~12.7)	15.1 (6.2~37.0) ^a#^	56.2 (23.4~95.3) ^b#,c#^	0.000
LDH, IU/L	334 (272~438)	440 (348~591) ^a#^	678(542~944) ^b#,c#^	0.000
PAB, g/L	0.11 (0.10~0.14)	0.10 (0.08~0.13) ^a#^	0.08 (0.06~0.12) ^b#,c#^	0.000
Subpopulations of T lymphocytes, %				
CD_3_	65.55 (58.48~74.84)	66.68 (59.92~74.23)	63.55 (54.74~72.39)	0.156
CD_4_	36.98 (31.64~43.66)	36.15 (29.90~42.09)	28.23 (23.76~36.60) ^b#,c#^	0.000
CD_8_	20.92 (16.70~26.28)	23.33 (19.44~28.99) ^a#^	26.48 (22.95~33.09) ^b#,c#^	0.000
Total Immunoglobulin (Ig)				
IgG, g/L	8.80 (7.10~10.73)	8.95 (7.60~10.70)	8.35 (7.18~10.63)	0.475
IgA, g/L	1.02 (0.69~1.37)	1.28 (0.87~1.78) ^a#^	1.25 (0.99~1.64) ^b#^	0.000
IgM, g/L	1.57 (1.16~2.03)	1.65 (1.22~2.24)	1.55 (0.99~2.40)	0.229
IgE, IU/ml	96.9 (37.6~218.5)	93.9 (38.4~290.5)	99.9 (51.1~287.3)	0.855
Cytokines, pg/ml				
IL-2	1.4 (1.1~1.7)	1.4 (1.1~1.7)	1.6 (1.1~2.4)	0.190
IL-4	2.0 (1.7~2.3)	2.0 (1.6~2.5)	2.2 (1.4~2.8)	0.593
IL-6	16.9 (7.9~43.4)	26.7 (13.3~57.8) ^a*^	59.5 (24.2~120.3) ^b#,c#^	0.000
IL-10	5.5 (4.4~7.6)	8.5 (6.0~12.0) ^a#^	9.4 (7.1~16.0) ^b#,c*^	0.000
TNF-α	2.5(1.8~4.6)	2.3(1.8~4.1)	1.7 (1.0~2.6) ^b#,c#^	0.000
IFN-γ	3.2 (2.2~4.8)	5.1 (2.8~10.8) ^a#^	8.4 (4.0~40.1) ^b#,c*^	0.000
D-dimer, mg/L	0.37 (0.25~0.44)	1.26 (0.82~2.07) ^a#^	7.68 (6.22~14.16) ^b#,c#^	0.000

Data are presented as the median (25^th^-75^th^ percentile). *P < 0.05, ^#^P < 0.01; ^a^compared between normal group and mild-moderately elevated group; ^b^compared between normal group and severe elevated group; ^c^compared between mild-moderately elevated group and severely elevated group. CRP, C-reactive protein; LDH, Lactate dehydrogenase; PAB, Prealbumin; IL-2, Interleukin 2; IL-4, Interleukin 4; IL-6, Interleukin 6; IL-10, Interleukin 10; TNF-α, Tumor necrosis factor-alpha; IFN-γ, Interferon-gamma.

### Radiographic Features of Patients

As shown in **Table 3**, we found that there were significant differences in radiographic features among the three groups (*P*<0.01). With the increase of D-dimer, the incidences of pleural effusion (26.4% *vs.* 64.2% *vs.* 93.5%, *P*<0.01), lobar atelectasis (3.8% *vs.* 33.3% *vs.* 63.0%, *P*<0.01), pulmonary consolidation (22.6% *vs.* 57.8% *vs.* 87.0%, *P*<0.01), large lesions (18.9% *vs.* 33.5% *vs.* 55.6%, *P*<0.01) were all significantly increased. As for necrotizing pneumonia, we found that it was likely to occur in the severely elevated group (23.9%, *P*<0.01), and there was no difference between the normal group and the mild-moderately elevated group (0.9% *vs.* 3.4%, *P*>0.05). However, none of the patients developed embolism in our study.

**Table 3:** Radiographic features of patients with MPP.

Radiological features, n (%)	Normal group (n=106)	Mild-moderately elevated group (n=204)	Severely elevated group (n=46)	*P*-value
Pleural effusion	28 (26.4%)	131 (64.2%) ^a#^	43 (93.5%) ^b#,c#^	0.000
Lobar atelectasis	4 (3.8%)	68 (33.3%) ^a#^	29 (63.0%) ^b#,c#^	0.000
Pulmonary consolidation	24 (22.6%)	118 (57.8%) ^a#^	40 (87.0%) ^b#,c#^	0.000
Large lesions	20 (18.9%)	66 (33.5%) ^a#^	30 (55.6%) ^b#,c#^	0.000
Necrotizing pneumonia	1 (0.9%)	7 (3.4%)	11 (23.9%) ^b#,c#^	0.000
Embolism	0 (0.0%)	0 (0.0%)	0 (0.0%)	1.000

Data are presented as number (percentage). ^#^P < 0.01; ^a^compared between normal group and mild-moderately elevated group; ^b^compared between normal group and severely elevated group; ^c^compared between mild-moderately elevated group and severely elevated group.

Large lesion was defined as the extent of infiltration on chest imaging more than 1/3 of the lung.

### Treatment of Patients

In addition to antibiotics, patients received other treatments depending on the disease severity, such as oxygen therapy, glucocorticoid, bronchoscopy, immunoglobulin, thoracentesis, or ICU admission (as shown in **Table 4**). In our study, we found that patients in the severely elevated group received a higher proportion of oxygen therapy, glucocorticoid, bronchoscopy, immunoglobulin, thoracentesis, and ICU admission when compared with the other two groups (*P*<0.01). Meanwhile, the usage rates of glucocorticoid, bronchoscopy and thoracentesis were likewise higher in the mild-moderately elevated group than those in the normal group (*P*<0.01). None of the patients needed mechanical ventilation. All the children recovered and were discharged from the hospital without death.

**Table 4:** Treatments of patients with MPP.

Treatments, n (%)	Normal group (n=106)	Mild-moderately elevated group (n=204)	Severely elevated group (n=46)	*P*-value
Oxygen therapy	28 (26.4%)	46 (22.5%)	24 (52.2%) ^b#,c#^	0.000
Glucocorticoid	51 (48.1%)	150 (73.5%) ^a#^	44 (95.7%) ^b#,c#^	0.000
Bronchoscope	21 (19.8%)	110 (53.9%) ^a#^	40 (87.0%) ^b#,c#^	0.000
immunoglobulin	3 (2.8%)	10 (4.9%)	14 (30.4%) ^b#,c#^	0.000
ICU	0 (0.0%)	2 (1.0%)	8 (17.4%) ^b#,c#^	0.000
Mechanical ventilation	0 (0.0%)	0 (0.0%)	0 (0.0%)	1.000
Thoracentesis	0 (0.0%)	15 (7.4%) ^a#^	19 (41.3%) ^b#,c#^	0.000

Data are presented as number (percentage). ^#^P < 0.01; ^a^compared between normal group and mild-moderately elevated group; ^b^compared between normal group and severely elevated group; ^c^compared between mild-moderately elevated group and severely elevated group.

### Correlation Analysis of the Level of D-dimer With Different Variables

**Figure 1:**
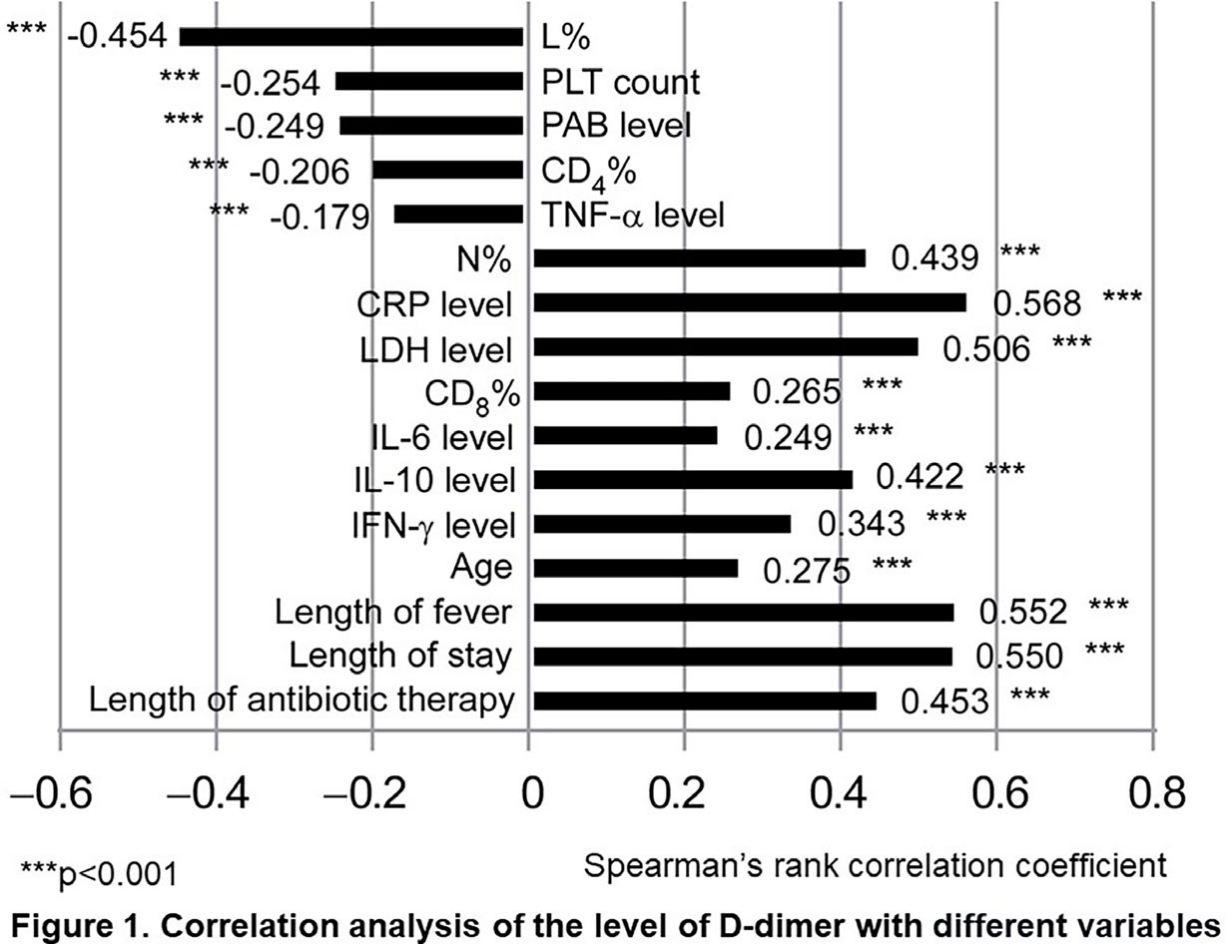
Correlation analysis of the level of D-dimer with different variables. Spearman rank-correlation coefficients were used to describe the association between different variables and D-dimer. Statistical significance was defined as P < 0.05, ***P < 0.001. N, neutrophil; L, lymphocyte; PLT, Platelet; CRP, C-reactive protein; LDH, Lactate dehydrogenase; PAB, Prealbumin; IL-6, Interleukin 6; IL-10, Interleukin 10; TNF-α, Tumor necrosis factor-alpha; IFN-γ, Interferon-gamma.

Using the Spearman correlation test, we analyzed the relationships between the level of D-dimer and different variables. As shown in **Figure 1**, we found that the following variables showed significant positive correlations with the level of D-dimer (*P*<0.01): N%, CRP, LDH, CD_8_^+^%, IL-6, IL-10, IFN-γ, age, length of fever, length of stay, and length of antibiotic therapy. Meanwhile, L%, PLT, PAB, CD_4_^+^%, and TNF-α were negatively correlated with the level of D-dimer (*P*<0.01). Among these variables, N%, L%, CRP, LDH, IL-10, length of fever, length of stay, and length of antibiotic therapy had particularly strong correlations with D-dimer.

## Discussion

*Mycoplasma pneumoniae* (MP) is one of the most common pathogens of CAP in children. In some cases, MP infection will cause severe pneumonia with a variety of complications (Poddighe, 2018). Recently, a few researchers have also reported that thrombotic diseases could occur in patients with MPP, which is thought to be associated with the elevated level of D-dimer (Li et al., 2017; Liu et al., 2020; Mirijello et al., 2020). The elevation of D-dimer is widespread in patients with MPP, but the incidence of thrombosis is not high, and no case of embolism was found, as well as in our study. Furthermore, to the best of our knowledge, there is little published literature focusing on the significance of elevated D-dimer in patients with MPP. So, in the present study, we retrospectively enrolled 356 MPP patients with different degrees of D-dimer and analyzed the relationship between the levels of D-dimer and clinical characteristics.

In our study, more severe signs and symptoms, higher incidence of extra-pulmonary complications, RMPP, SMPP, and longer process of disease were found in the elevated groups, especially in the severely elevated group. And there were more severe pulmonary lesions and higher levels of inflammatory biomarkers, such as CRP, LDH, N%, CD_8_^+^%, IL-6, IL-10, and IFN-γ, which were in line with the increase of D-dimer, whereas the L% and PAB decreased with the levels of D-dimer. Furthermore, we found that N%, L%, CRP, LDH, IL-10, length of fever, length of stay, and length of antibiotic therapy had strong correlations with D-dimer.

D−dimer was originally known as a specific fibrin degradation product, as well as a specific marker of the fibrinolytic system, which can reflect the coagulation function and fiber activity of the body (Zhang et al., 2020). However, recently, it has also been recognized as an indicator for evaluating the severity of CAP (Snijders et al., 2012). The results in our study showed that MPP patients with higher levels of D-dimer had more severe clinical manifestation and needed a longer duration of treatment, which might help to confirm other research’s finding that D-dimer could act as an indicator for evaluating the severity of disease.

Cell-mediated immunological response plays a major role in the progression of MPP (Waites et al., 2017). Our preceding study and several other studies have demonstrated that inflammatory cytokines and some serum biomarkers were involved in the immunopathogenesis of MP infection (Tanaka et al., 2002; Narita and Tanaka, 2007; Lu et al., 2015; Choi et al., 2019; Ling et al., 2020). Furthermore, with the progress of MPP, immune cells release different kinds of inflammatory mediators, such as IL-1β, IL-8, TNF-α, and then aggravate the injury of vascular endothelial cells, leading to a significant increase of D-dimer (Iturriaga et al., 2015; Mishra et al., 2015; Jin et al., 2018). In this study, we found that some cytokines and inflammatory markers (CRP, LDH, N%, CD_8_^+^%, IL-6, IL-10, and IFN-γ) were significantly increased in the elevated groups, and there were highly positive correlations between the levels of D-dimer and some inflammatory markers (N%, CRP, LDH, IL-10). These results implied that higher levels of D-dimer might be associated with stronger inflammation, which was consistent with other reports (Yu et al., 2020).

MP is well recognized for producing a broad array of extra-pulmonary manifestations. More interestingly, our study found that the incidence of extra-pulmonary complications was much higher in the elevated groups than that in the normal group, and the incidence of extra-pulmonary complications was increased with the level of D-dimer. To our knowledge, this is the first report showing the relationship between the level of D-dimer and the extra-pulmonary complications in MPP patients. The mechanisms of MPP causing extra-pulmonary complications, by far, are not fully understood. Some studies have shown that it might be a direct effect of the MP that presents at the site of inflammation mediated by cytokine release by the host (Waites et al., 2017). Some researchers considered that it might be a direct or indirect effect by the production of vasculitis or thrombosis as a result of cytokines and chemokines or by immunomodulation through mediators, such as complement and fibrin D-dimers (Narita, 2009; Li et al., 2017; Waites et al., 2017). The latter might be the underlying mechanisms of the phenomenon in our study that the higher the incidence of extra-pulmonary complication was, the higher the D-dimer value was. Recent medical literature also suggested that increased IgE levels (atopy) might be associated with extra-pulmonary manifestations in children with MPP (Wang et al., 2019). However, we found that there were no significant differences in IgE levels among all three groups in our study. This might be because that different age ranges of patients among groups caused different normal ranges of IgE.

There were several limitations in our study. First, our study was a single-center retrospective study, which might have introduced a selection bias. The results reported in our study cannot be extrapolated to other areas of China. Thus, a prospective multicenter study is needed in the future. Second, there might be some patients who had coinfection with other pathogens, which could not be detected precisely and might therefore lead to the elevation of D-dimer. Third, we did not monitor dynamic changes of D-dimer. Therefore, it may result in some omissions of hypercoagulable states.

In conclusion, MPP patients with higher levels of D-dimer might have more severe clinical manifestation and need a longer duration of therapy, which was perhaps closely related to the degree of inflammation of MPP. These findings might help physicians to have deeper insights into MPP and provide proper treatment for MPP patients with higher levels of D-dimer.

## Data Availability Statement

The original contributions presented in the study are included in the article/supplementary material. Further inquiries can be directed to the corresponding authors.

## Author Contributions

Conceived and designed the experiments: YYZ and ZMC. Wrote the manuscript: YZ and YYZ. Collected and analyzed the data: YZ, LLH, QNZ, MYL, YSW, YLZ and MXH. All authors contributed to the article and approved the submitted version.

## Funding

This work was supported by grants from National Natural Science Foundation (81871264).

## Conflict of Interest

The authors declare that the research was conducted in the absence of any commercial or financial relationships that could be construed as a potential conflict of interest.
